# Ergot in the Treatment of Fibroid Tumors of the Uterus

**Published:** 1879-10

**Authors:** W. H. Byford

**Affiliations:** Prof. of Gynæcology in Rush Medical College


CHICAGO MEDICAL

JOURNAL & EXAMINER.

VOL. XXXIX.—OCTOBER, 1879.—NO. 4.

[In these pages dimension and weight are expressed in terms of the Metric System, and temperature in degrees of the Centigrade Scale.]

### Original Communications.

### ARTICLE I.

ERGOT IN THE TREATMENT OF FIBROID TUMORS OF THE UTERUS. BY W. H. BYFORD, A.M., M.D., Prof. of Gynæcology in Rush Medical College. Read before the Obstetric Section of the British Medical Association.

As an introduction to my paper, I submit the following propositions: 1st, When properly administered, ergot frequently very greatly ameliorates some of the troublesome and even dangerous symptoms of fibroid tumors of the uterus, *e. g.*, hæmorrhage and copious leucorrhœa; 2d, it often arrests their growth and checks hæmorrhage; 3d, in many instances it causes the absorption of the tumor, occasionally without giving the patient any inconvenience, at other times the removal of the tumor by absorption is attended by painful contractions and tenderness of the uterus; 4th, by inducing uterine contraction it causes the expulsion of the polypoid variety of the submucous tumor; 5th, in the same way it causes the disruption and discharge of the intramural tumor.

There are many cases on record to substantiate every one of these propositions.

From what I consider well-authenticated sources, including the cases under my own observation and in the practice of my friends and neighbors, I have collected one hundred and thirty-six cases of fibroid tumors treated by ergot. Of these, twenty-five cases were cured without giving the patient any inconvenience from painful contractions. In forty-six cases the tumors were diminished in size and the hæmorrhage was cured. In twenty-seven others the hæmorrhagic symptom was relieved, while the size of the tumor was not affected. In eight other instances the tumors were broken to pieces and expelled piece-meal.

For examples of cases in which the first conditions obtained, I would refer to those cured by Hildebrandt; of the other examples, four were reported to me by Dr. S. P. White, of Buffalo, U. S., one by the late Dr. Hodder, of Canada, and Dr. Jukes each, and eleven occurred among my immediate acquaintance and in my own practice.

Among those in which the hæmorrhage was cured and a diminution of the tumor took place, eleven occurred to Hildebrandt, two to Chrobak, five to White, of Buffalo, and the remainder to gentlemen upon whose veracity I have implicit reliance. The most remarkable case of which I have any knowledge was reported to me by the late Dr. G. C. Goodrich, of Minneapolis, U. S., in which absorption of a large tumor took place under the administration of ergot and belladonna. I subjoin his description:

"The treatment was commenced in 1870, and continued two years. The uterus filled the whole space between the ilia, and measured in the transverse diameter thirty ctm., and in the vertical forty-eight ctm., extended up under the ensiform cartilage and close up to the margin of the cartilages of the ribs. The treatment was followed by cramps in the uterus, which produced a wild enthusiasm in the mind of the patient, and inspired her with strong hopes of recovery. Without consulting me, she doubled the dose of medicine, which was administered internally, and as a consequence she was attacked with very strong uterine contractions and symptoms of metritis. This caused me to abandon treatment for about one month, and had it not been for the urgent determination of the patient, I would not have resumed it. She insisted that as this was the first medicine which had ever affected the enlarged organ, she believed it would cure her, and promised to obey my directions if I would proceed. She so promptly and rapidly improved that I doubted if it were not a coincidence with, rather than a consequence of, the treatment. Prompted by this doubt, I abandoned the use of the ergot and belladonna, and continued alterative treatment. The patient soon assured me that she no longer felt the griping pains caused by the remedy, and that the tumor was softer and larger than when she took the ergot prescription. The ergot and belladonna were again resumed, and in four months she was able to make a trip to Boston alone. While absent, she continued to take the medicine. From this time she continued rapidly convalescing, and is now in the enjoyment of fine health."*

I subjoin two cases in which the tumors were expelled piecemeal, under the administration of ergot, which came under my own observation :

Mrs. Arthur King, of Sterling, Illinois, called on me December 13, 1875. She was thirty-five years old, married, and had never been pregnant.

On the first of the preceding June she noticed a circumscribed hard lump two inches below, and to the left of the umbilicus. She was the subject of serious uterine and sympathetic symptoms, for which she had at different times had treatment. She had profuse menorrhagia, leucorrhœa, and great sense of weight in the pelvis.

Upon examination, I found a hard, round, movable tumor, extending up to within five ctm. of the umbilicus, filling up the whole of the right iliac, the hypogastric, lower half of the umbilical, and more than half of the left iliac regions.

The contour of the tumor was somewhat uneven, though not distinctly nodular. The cervix was long, pointed, and thrown backward and to the left. The sound entered the small uterine mouth and passed upward, backward, and to the left fourteen centimetres.

* The author's address before the Am. Med. Association, at its meeting in 1875.

The diagnosis was a fibrous tumor of the right anterior wall of the uterus. I prescribed thirty drops of Squibb's fluid extract of ergot to be taken three times a day. She went home, but did not commence taking the medicine until the 20th of December. On the 26th of December Dr. J. B. Crandall was called to see her, and describes her condition as follows: "The patient was in a state of great nervous prostration, and worn out by severe pain and loss of sleep. The pains commenced soon after taking the second dose of ergot, and were excruciatingly severe for about three hours, after which they continued less severely for two days and nights. She had more or less hæmorrhage from the uterus after taking the ergot. Her pulse was feeble, 110 to 120 to the minute. The skin was hot and dry, and she complained of great pain and tenderness over the uterus and lower bowels. The feet were drawn up, and the face wore a pinched and peculiar expression." Under these circumstances, the doctor administered anodynes, tonics and nourishment, to the great relief of the patient.

On January 11, 1876, the patient began to pass from the vagina small masses of fibrous substance, from the size of a chestnut to that of an English walnut. The substances thus discharged were firm, and gray in color, and were exceedingly fetid. This discharge continued up to the 21st of January, when the uterus was very much diminished in size, the tenderness had subsided, and the patient appeared comparatively comfortable. Up to that time she had taken but three doses of ergot — on the 20th of the preceding month — and the doctor ordered it to be resumed again. This time the ergot produced no pain, and after three or four days was discontinued. From the 21st of January there were no more pieces discharged, but up to February 1st a yellowish, thin, offensive fluid passed from the vagina in considerable quantities. On the first day of February the ergot was again ordered and continued two weeks, when, as no results ensued, it was finally dropped.

Dr. C. states that on the 14th of February the uterus was reduced to its normal size, and on the 26th the patient was up and about her work, completely cured. He remarked, in this connection, that the first three doses of ergot taken by the patient were the cause of her recovery.

This case is published in the August (1875) number of the CHICAGO MEDICAL JOURNAL AND EXAMINER, as reported by Dr. Crandall.

Mrs. L. D. M., age 47 years, had a fibroid tumor in the anterior wall of the uterus, which, with the enlarged uterus, arose to within five ctm. of the umbilicus.

She commenced taking thirty drops of the fluid extract of ergot on the 22d of September, 1876, and was to increase gradually the dose with the object in view of causing the disruption and expulsion of the tumor. The ergot at first produced no perceptible effect, until she had taken it ten days, when she began to experience the pain of contraction. The pain became so severe and continuous that it was necessary to omit it for two or three days at a time. The patient was intelligent and understood the object and mode of action of the ergot, and when the pain entirely subsided, she courageously resumed it in the smaller doses, and increased again until the pains became intolerable. On the 13th of January, 1877, small pieces of the tumor showed themselves in the vaginal discharges, and by the 26th of the same month the whole of it had been discharged piece-meal.

She wrote me on the 30th of January, saying, "I think I wrote one week ago to-day. At that time the tumor was passing. It continued to pass until the 26th, when, I think, the last was expelled. To-day I send you by express a portion of the last that came. I think the whole of it including the portion I sent you, would have weighed one and a half pounds. I do not believe a quart can would hold it if the whole had been preserved. It commenced to come on Saturday, and from Saturday evening to Sunday morning, there was a pint or more. After that the stench was so disagreeable that we could not cleanse it; consequently we threw it away. Wednesday and Thursday it seemed to be in one continuous mass. I cannot better describe it than to say that it came like sausage-meat, from a stuffer. I would cut off about four inches a day, that is on Wednesday and Thursday. On Friday morning the last of it came away."

During, and for some days after, the expulsion, she suffered slight symptoms of septicæmia, but recovered from them, and in the course of a month afterward she visited me, when I found the uterus measured six centimetres in depth. She then had some leucorrhœa, but was fast regaining her health. She is now perfectly well, and has passed in safety the menopause.*

I have known eight cases in which the tumors were expelled piece-meal by ergot, with but one death. The death occurred in a patient who rode one hundred and fifty miles on a railroad train to see me, with pieces of the tumor hanging from the vagina, which she would not allow her physician to remove. When she arrived, I passed my fingers up into the contracted capsule and scooped out the remaining portion of the tumor. She was so exhausted, however, by the journey and the sepsis that she died three days afterwards.

I cannot help believing that if she had remained at home and submitted to the treatment of her physician, her life need not have been sacrificed.†

The influence of ergot over the uterus has been a familiar fact to the profession for a long time. It is not long, however, since we were aware of its effects upon the muscular fibers entering into the formation of other organs. We now know that this medicine acts upon the unstriped muscular fiber wherever found, whether in the viscera or in the vessels of the body.

The fibers of the uterine walls, and the arteries supplying them with blood, both belong to this class ; this fact in the formation of the uterus renders it particularly susceptible to the action of ergot. This drug acts upon the uterus‡ in a threefold manner, and causes a diminished flow of blood to the morbid as well as healthy tissues in the uterine structure.

First : the calibre of the arterial tubes is diminished by the contraction of the muscular fibers which enter into their composition. Second : the arterioles are diminished in size by compression from the contraction of the uterine muscular fibers which surround them. Third : these vessels are distorted and drawn in diverse directions by both the contraction and compression, and hence are rendered less fit for sanguineous conduits.

---

* This case — the abstract of which I have here given — was published in the May 15th, 1877, No. of the *Archives of Clinical Surgery*, N. Y.

† This case was published in the May 15th, 1877, No. of the *Archives of Clinical Surgery*, New York.

‡ From the author's address before the Am. Med. Association, 1875.

Another consideration of prime importance is that, under the influence of these medicines, the nutrition of fibrous tumors is interfered with, not only from diminution of blood in their tissues, but also from compression of their substance by the proper fibers of the uterus, and are therefore made more susceptible to the process of disintegration and absorption.

The great influence exerted by ergot over the circulation of the uterus is rendered more efficacious in the removal of fibroid tumors of that organ, because of the peculiar organization of the growths. It is now pretty well understood that this neoplasm is not very generously supplied with arterial blood, and that its supply is derived from numerous minute vessels instead of one or two of larger caliber. From these circumstances it results that its vitality is very low, its circulation easily disturbed, and consequently its nutrition impaired.

I think we are justified from observation in assuming that the action of ergot may be graded from an almost imperceptible to a very intense degree. Probably the first degree effects the vascular supply; the second, in addition to this, causes so much contraction as to merely render the fibers tense without causing pain; and the third prompts the uterine fibers to vigorous and painful contraction.

This inference is plainly deducible, I think, from the several modes by which tumors are made to disappear under its action, as well as from direct observation of the uterine fibers.

Before going further, I desire to allude briefly to the relations of fibroid tumors to the different strata of the muscular fibers of the uterus. We use the terms *submucous*, *intramural* and *subserous*, as expressive of this relation. These terms are too vague to carry with them a correct idea of the facts, without some explanation. *All fibroid* tumors of the uterus have their origin in the wall of that organ. Some arise immediately in contact with the mucous membrane, and, as they grow, become pediculated and hang in the uterine cavity. Others commence their growth beneath a very thin layer of fibers. They very soon overcome the resistance of the attenuated stratum, and pushing it and the mucous membrane before it, become pediculated later in their growth.

If, however, they are deeper in the wall, but between the central layer of its fibers and the mucous membrane, their bulk gradually encroaches upon the interior of the uterus, forming broad tumors that fill the cavity of the organ.

These are not attached to, but developed *in* the wall of the uterus. These are all submucous tumors, but in professional language the two first are called polypoid, while to the latter the term submucous tumor is given. We designate the tumor that is developed exactly in the central layer of the wall of the uterus, displacing the surrounding tissues alike in every direction, intramural. In point of fact this variety of tumor is very rare, as the fibroid growth much more frequently originates external or internal to the central line of the wall. The subserous tumor varies in its relative distance from the peritoneum in the same manner as the submucous from the lining membrane of the uterus.

I will now venture to call attention especially to the manner of expulsion of the polypoid and submucous intramural varieties. It will be seen from figure No. 1 that when the uterus contracts, all the fibers unite in pressing the polypus through the cervical canal, which is usually already shortened, and rendered dilatable in consequence of its increased vascularity.

The cervical canal dilates, and after more or less painful efforts the polypus is expelled entire, covered by the mucous membrane. This membrane is often in a state of gangrene, but so far as I have observed these cases, the tumor is not broken to pieces.

Figure 2 represents an intramural fibroid between the central line of the uterine wall and the mucous membrane. It is intended to show a tumor where a thin layer of fibers separates it from the mucous membrane, and where a thick and heavy layer is spread over its external hemisphere. Three quarters of the muscular wall are applied to that side of the tumor. If in this position all the fibers of the uterus vigorously contract, the fibers near the mucous membrane must be overcome by the heavy layer outside (at *c*). But the opposite wall plays an important part by supporting the weaker layer at the fundus of the tumor, and adding its own force in overcoming the capsule at (*e*), where it usually gives way. The position of the tumor makes its escape from the concentric action of all the fibers of the uterus impossible, and every one knows that when the resistance is partially overcome, the uterus is stimulated to more vigorous action, and the pains will not abate until the mass is expelled. If not too large, it is driven out without undergoing great laceration, but if its size and attachments are such as to make this impracticable, it will be broken into fragments and expelled piece-meal.

Allow me to supplement the above description by explaining the effect of ergot on the sub-peritoneal and central intramural tumor. In figure 3, we see the disposition of the fibers on the sub-peritoneal variety, next the uterine cavity there is a thick and strong stratum of fibers, while immediately under the peritoneum the layer is very thin and comparatively weak. When the uterus is acting with vigor, the fibers between A and B, will cause those two points to approximate each other, and the tumor will become pediculated; but that is all, for the tumor lays outside the field of concentric action and escapes the crushing influence to which the submucous variety is subjected. The amount of force exerted upon it is that exercised by the weaker layer of fibers in a state of conquered antagonism, and the rupture of the capsule is impossible.

If we take figure 4 as a correct representation of the fibroid tumor when situated in the central stratum of fibers, in which the antagonism is equal at all points, it will be evident that there is no tendency to rupture of the capsule, and much less crushing influence exerted upon it than if it were situated slightly nearer the mucous membrane.

This variety of the tumor, therefore, yields to the influence of ergot, only as it may be "starved out" by diminution of its blood supply, and as the effect of pressure, which we all know are the two conditions most favorable to absorption.

Now I think we have arrived at a point in this investigation where we can draw inferences as to the forms of tumors likely to be effected by ergot in different ways, as well as those that will not be effected by it.

We do not expect ergot to cause painful and efficient contractions in the healthy unimpregnated uterus; its fibers are not capable of such contraction, and it is not until the fibers have become greatly developed that they are susceptible to the impressions of ergot. In cases of early abortion, its action is very unreliable, but after the fourth month of pregnancy it acts quite efficiently.

In tumors of the uterus, the development of the fibrous structure is sometimes so slight that it is incapable of contraction ; there may be so many nuclei of degeneration that there are not enough sound fibers left for efficient contraction. Then, where there are many small tumors developed in the uterine walls, the circulation is cut off to such a degree that they degenerate into a cartilaginoid substance, and sometimes they are infiltrated with calcareous material. In none of these cases will ergot cause any appreciable results. When, however, there are but one, two or three nuclei of morbid growths, as they increase in size the fibers undergo the development necessary to enable them to contract with great efficiency, and render them susceptible to the influence of ergot.

Another condition which influences the hypertrophic growth of the fibers is the situation of the tumor.

Sub-peritoneal tumors do not cause as great growth in the fibers of their neighborhood as the intramural or submucous varieties. A single intramural tumor causes great development of the whole uterine tissues, but the development of the wall in which it is situated decidedly predominates. The submucous neoplasm so soon gains the uterine cavity, that the development is nearly the same in the whole organ.

When, therefore, we administer ergot for the cure of fibrous tumors of the uterus, the beneficial action of the drug will depend upon the degree of development of the fibers of the uterus, and the position of the tumor with reference to the serous or mucous surface. The nearer the mucous surface, the better the effects. If the tumor is very near the lining membrane, we may hope for its expulsion *en masse*, or by disintegration.

We can often select the cases in which good results may be expected. There are four conditions which are usually reliable for this purpose. They are, smoothness of contour, hæmorrhage, lengthened uterine cavity, and elasticity. A smooth, round tumor denotes, for the most part, uniform textural development; hæmorrhage, a certain proximity to the mucous membrane; a lengthened cavity, great increase in the length and strength of the fibers ; and elasticity assures us of the fact that cartilaginoid or calcareous degeneration has not begun in the tumor.

An even nodulated tumor may be composed of many separate solid masses. These displace and prevent the growth of the fibers to such an extent as to render contractions inefficient. When hæmorrhage is not present the tumor is probably near the serous surface, and consequently not surrounded by fibers. A short cavity denotes short, undeveloped fibers; while hardness is indicative of unimpressable induration.

Although I have no experience in the use of ergot in such cases, I should expect large fibro-cystic tumors to resist the action of ergot.

From this view of the subject it will be seen that I freely admit that there is a large number of cases in which ergot cannot produce any good results, in consequence of the nature of the cases; but there is another reason of equal moment why ergot may fail to act upon such cases as would seem to be favorable, by the worthlessness of the drug and its preparations.

Dr. Squibb, of New York, a high authority, says in reference to this subject:

"The molecular constitution of the active portion of the drug seems, however, in its natural condition to be loose, and like a slow fermentation, to be undergoing slow molecular changes, so that by age its peculiar activity is slowly diminished until finally lost."

And again, "The ergot in the grain, however well kept, is known to become inactive without any known change in appearance, though the sensible properties, such as odor and taste, may and probably do not change. Ergot, in powder, is known to diminish in activity much more rapidly than when in grain, and probably soon becomes inert. The tincture and wine of ergot are believed to change, though more slowly than the ergot in substance. Whilst the extracts, and so-called ergotins, are all supposed to change more rapidly."

These facts, so explicitly stated by Dr. Squibb, are very suggestive as to the causes of the frequent failures of ergot, and need no comment.

When all these causes of failure are considered, the variety of experience met with in the reports upon its trial in the treatment of these tumors is not surprising. It should not, however, be discouraging; but should prompt us to more care in selecting the cases and securing reliable preparations of ergot. I have implicit faith in the action of ergot when all the conditions I have pointed out are present. I do not believe it to be uncertain in its action.

In addition to the above conditions, I believe perseverance an indispensable condition to success, as it often requires several months to get the best results.

The mode of administration should be governed by the objects to be attained. If we desire to cause the painless absorption of the tumor, the doses ought to be moderate in size, and not too frequently administered. Hildebrandt administered by hypodermic injection a preparation in quantities which represented from fifteen to twenty grains of the crude drug once daily, or once every other day; and it will often be sufficient — as proven by cases quoted in my address above quoted — once a week. If we desire to have the tumor expelled, we should administer full and increasing doses often repeated, and continued until the object is attained.

It will sometimes be necessary to vary the quantity and times of giving it to suit the susceptibility of the patient; less or more according to the amount of pain caused by it.

It is not essential to give it hypodermically, although when it does not produce much inconvenience this is a very efficacious method, it may be given by the mouth, in suppositories, per rectum, etc.

In concluding my paper, I desire to disclaim any expectation that ergot will supplant other modes of treatment. The expert surgeon will, as he always has, use his instruments to the neglect of remedies less summary in their effects, and in his hands the maximum of safety will obtain, but there are very few general practitioners, who ought or would be willing to undertake enucleation of fibroid tumors of the uterus. I do claim, however, that the judicious gynæcologist will lose fewer patients and make more cures by the consistent administration of this medicine, than can be looked for from surgery.

